# The Cross-Species Immunity During Acute *Babesia* Co-Infection in Mice

**DOI:** 10.3389/fcimb.2022.885985

**Published:** 2022-05-27

**Authors:** Iqra Zafar, Eloiza May Galon, Daisuke Kondoh, Artemis Efstratiou, Jixu Li, Shengwei Ji, Mingming Liu, Yongchang Li, Yae Hasegawa, Jinlin Zhou, Xuenan Xuan

**Affiliations:** ^1^National Research Center for Protozoan Diseases, Obihiro University of Agriculture and Veterinary Medicine, Obihiro, Japan; ^2^Livestock and Dairy Development Department, Veterinary Research Institute, Lahore, Pakistan; ^3^Department of Veterinary Medicine, Obihiro University of Agriculture and Veterinary Medicine, Obihiro, Japan; ^4^Max Planck Institute for Evolutionary Biology, Plön, Germany; ^5^College of Agriculture and Animal Husbandry, Qinghai University, Xining, China; ^6^Department of Microbiology and Immunology, School of Basic Medicine, Hubei University of Arts and Science, Xiangyang, China; ^7^Parasitology Laboratory, Veterinary College, Xinjiang Agricultural University, Urumqi, China; ^8^Shanghai Veterinary Research Institute, Chinese Academy of Agricultural Sciences, Shanghai, China

**Keywords:** *Babesia microti*, *Babesia rodhaini*, acute stage, co-infection, babesiosis, tick-borne infection, innate immunity, oxidative stress

## Abstract

Babesiosis causes high morbidity and mortality in immunocompromised individuals. An earlier study suggested that lethal *Babesia rodhaini* infection in murine can be evaded by *Babesia microti* primary infection *via* activated macrophage-based immune response during the chronic stage of infection. However, whether the same immune dynamics occur during acute *B. microti* co-infection is not known. Hence, we used the mouse model to investigate the host immunity during simultaneous acute disease caused by two *Babesia* species of different pathogenicity. Results showed that *B. microti* primary infection attenuated parasitemia and conferred immunity in challenge-infected mice as early as day 4 post-primary infection. Likewise, acute *Babesia* co-infection undermined the splenic immune response, characterized by the significant decrease in splenic B and T cells leading to the reduction in antibody levels and decline in humoral immunity. Interestingly, increased macrophage and natural killer splenic cell populations were observed, depicting their subtle role in the protection. Pro-inflammatory cytokines (i.e. IFN-γ, TNF-α) were downregulated, while the anti-inflammatory cytokine IL-10 was upregulated in mouse sera during the acute phase of *Babesia* co-infection. Herein, the major cytokines implicated in the lethality caused by *B. rodhaini* infection were IFN- γ and IL-10. Surprisingly, significant differences in the levels of serum IFN- γ and IL-10 between co-infected survival groups (day 4 and 6 challenge) indicated that even a two-day delay in challenge infection was crucial for the resulting pathology. Additionally, oxidative stress in the form of reactive oxygen species contributed to the severity of pathology during acute babesiosis. Histopathological examination of the spleen showed that the erosion of the marginal zone was more pronounced during *B. rodhaini* infection, while the loss of cellularity of the marginal zone was less evident during co-infection. Future research warrants investigation of the roles of various immune cell subtypes in the mechanism involved in the protection of *Babesia* co-infected hosts.

## Introduction

Babesiosis is an emerging tick-borne zoonotic disease caused by the intraerythrocytic parasite *Babesia*, resulting in a malaria-like disease (Dvoraková & Dvorácková, 2007). *Babesia rodhaini* is a rodent *Babesia* related to *B. microti*, the major causative agent of human babesiosis. Both species belong to the “small group” of *Babesia*, characterized by the relatively smaller size (1.0-2.5 µm) of trophozoites (Homer et al., 2000). *Babesia rodhaini* has the potential to infect human erythrocytes (Kawabuchi et al., 2005) and inoculation of even a single parasite leads to 100% mortality in mice (Clark, 2001). In contrast, *B. microti* (Munich strain) causes a self-limiting disease in mice that resolves eventually (Igarashi et al., 1999). *Babesia microti* causes babesiosis in animals and humans worldwide and has seen increasing interest as an emerging zoonosis  (Leiby, 2011; Vannier & Krause, 2012). *Babesia* is not only transmitted by tick vectors but also by blood transfusion or during pregnancy (Young et al., 2019; Xue et al., 2021). According to the CDC, approximately 2,000 cases of babesiosis occur every year in the United States (Centers for Disease Control and Prevention, 2012). *Babesia* is a menace to the supply of blood and is lately the most common transfusion-transmitted infection in the United States (Lobo et al., 2013). Babesiosis can be life-threatening in infants or immunocompromised patients (Gabrielli et al., 2016).

The spleen is the largest secondary lymphoid organ in the body and is involved in hematopoietic functions. It elicits immunological responses against blood-borne pathogens, rendering it indispensable in interrelating the immune system’s response (Cesta, 2006). The spleen is likewise involved in the clearance of parasitized erythrocytes by filtration, including babesiosis (White et al., 1998). Eventually, the fate of the host mainly relies on whether the host has an intact spleen (Roberts et al., 1972). Splenectomized and elderly individuals are at a higher risk of severe symptomatology and may experience hemolytic anemia, splenomegaly, hepatomegaly, renal failure, and even death (Bloch et al., 2019; Young et al., 2019). Hence, as the biggest immune organ, the spleen plays an essential role in protecting against *Babesia* infection. When infected with *Babesia*, even though the spleen is seriously injured, it still actively initiates immunomodulatory responses (Xue et al., 2021). The spleen consists of B lymphocytes and macrophages and can also produce immunoglobulins and factors that deploy immune functions (Golub et al., 2018). Some studies indicated that cellular immunity, T cells, and macrophages are critical for the clearance of *B. microti* in mice (Igarashi et al., 1999; Clawson et al., 2002; Li et al., 2012). Still, these studies are uncertain in establishing the crucial function of each of these factors in the resolution of infection (Skariah et al., 2017). No vaccine has yet proven effective against *Babesia* infection (Man et al., 2017). More importantly, the limited knowledge of the mechanisms of immunity and pathogenesis in babesiosis causes hindrance to the development of effective preventive and therapeutic interventions (Vos & Bock, 2006; Rathinasamy et al., 2019).

Co-infection, or mixed infection, is a type of infection wherein a host is concomitantly infected with two or more pathogens (Cox, 2001). Co-infections can be inconsequential, deleterious, or even beneficial, and have complex interactions, including modulation of the host response (McArdle et al., 2018). The actual clinical picture of concomitant infections involves diverse microorganisms that have an influence not only on each other but also on the host (Lundqvist et al., 2010). However, existing knowledge is limited on which co-infections are of importance to our health (McArdle et al., 2018). In the context of babesiosis, simultaneous infection with two Babesia species has been recorded in humans (Mayne, 2014), animals (Young et al., 2019), and tick vectors (Steiner et al., 2008). However, information concerning the prevalence of co-infections is scant (Moutailler et al., 2016). Unlike the *Plasmodium* species, the pathogenesis of *Babesia* infection and co-infections remains understudied; hence, it is poorly understood despite its significant impact on human health. Previously, immunity to babesiosis had been demonstrated in a phenomenon referred to as heterologous immunity. To the best of our knowledge, only a few studies have investigated *B. microti* and *B. rodhaini* co-infections experimentally using mouse models (Cox & Young, 1969; Zivkovic et al., 1984; Inoue et al., 1994; Li et al., 2012; Wang et al., 2016). *Babesia microti* provided cross-protective immunity against *B. rodhaini* (Li et al., 2012). During the chronic stage of the *Babesia* co-infection, significant decrease in antibody levels and blood cytokine levels in mouse sera were observed. In addition, macrophages were identified as the immune cell primarily responsible for the conferment of cross-species protection against a subsequent lethal challenge (Li et al., 2012). However, whether the same immune dynamics occur during acute *Babesia* co-infection is not known.

In the present study, we highlighted the importance of investigating concomitant acute co-infections and a better understanding of immune dynamics involved in the protective immunity induced post-*B. rodhaini* lethal challenge during acute primary babesiosis in mice.

## Materials and Methods

### Experimental Animals

Female wild-type (WT) BALB/c mice (6–8 weeks old, weighing 18–22 g) were purchased from CLEA Japan. Mice were housed in pathogen-free conditions as formerly described (Li et al., 2021). Female mice were used to eradicate interference that might arise due to variation in hormonal profile ultimately affecting parasitic interactions (Djokic et al., 2019).

### *Babesia* Parasites and Experimental Infections

**Figure 1:**
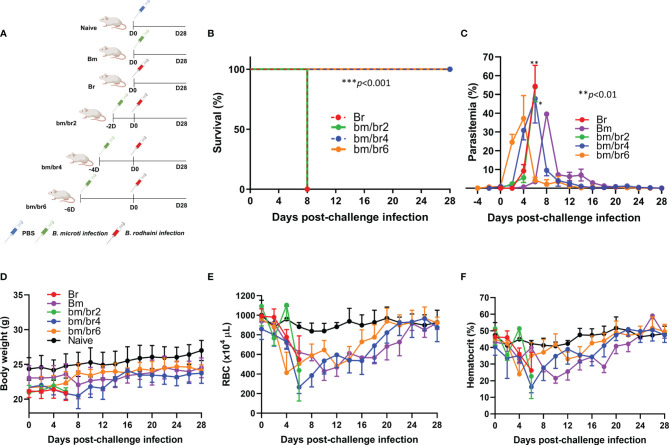
The course of *B. rodhaini* challenge infection in BALB/c mice undergoing acute *B. microti* infection. Test BALB/c mice were initially infected with *B. microti* and then challenge infected with *B. rodhaini* at different time points (on days 2, 4, 6) post-primary infection. **(A)** Overall experimental plan. **(B)** Survival curve, **(C)** course of parasitemia, **(D)** body weight, **(E)** red blood cell (RBC) count, and **(F)** hematocrit values were monitored for 28 days after challenge infection. Mean percent parasitemia, body weight, RBC, and hematocrit values were calculated from individual values taken from all surviving mice at each specific time point. Results are expressed as the mean values ± standard deviation (SD) of six mice (n = 6). Ordinary one-way analysis of variance (ANOVA) with Tukey’s test was used for the comparison of parasitemia between Br and co-infected groups, while the Kaplan-Meier non-parametric model was used for the survival analysis. Asterisks indicate statistical significance (*p < 0.05; **p < 0.01; ***p < 0.001).

*Babesia microti* (Munich strain) and *B. rodhaini* (Australia strain) frozen stabilates were taken out from the cell bank and intraperitoneal inoculations were performed to serially passage and maintain the parasites in mice *in vivo*. In the current study, infection trials were conducted to determine the effect of an initial *B. microti* infection on a subsequent infection. Initially, three groups (n=6 per group) were infected intraperitoneally with 10**^7^** *B. microti*-infected red blood cells (RBCs). These three groups were challenge infected with 10**^6^** *B. rodhaini*-infected RBCs at day 2 (hereafter referred to as bm/br2; n=6), day 4 (hereafter referred to as bm/br6; n=6), and day 6 (hereafter referred to as bm/br6). Similarly, a separate group (n= 6) was intraperitoneally inoculated with 10**^6^** *B. rodhaini*-infected RBCs only (hereafter referred to as Br; n= 6), another group with 10**^7^** *B. microti*-infected RBCs only (hereafter referred to as Bm; n=6), and an uninfected group (Naive; n=6) injected with phosphate buffered saline (PBS) only (**Figure 1A**). The first trial was performed to determine at what co-infection time point does cross-protection happen. Then, a second trial was conducted only including groups wherein cross-protection was observed from the first trial (bm/br4 and bm/br6), control groups for each parasite species (Br and Bm), and a group of naive mice to assess the immune dynamics during cross-protection.

### Assessment of Parasitemia and Survival Rates

Parasitemia, body weight, hematocrit values, and survival rates were regularly monitored every other day. Hematocrit values were measured as previously described (Wang et al., 2016). Approximately 10 μL of blood was withdrawn from the tail vein of the mouse and diluted 200 times with Isotonac 3 buffer (Nihon Kohden, Tokyo, Japan). The hematologic values were obtained using the Celltac Alpha MEK-6550K (Nihon Kohden). The percent parasitemia was determined by thin blood smears stained with Giemsa and calculated from 10^3^ erythrocytes examined under 100 × oil immersion Eclipse E200 microscope (Nikon, Tokyo, Japan).

### Immunofluorescence Microscopy Analysis (IFA)

Blood obtained from mice was diluted to 1:40 in PBS and washed three times. Blood smears were fixed on glass slides with ethanol/methanol (1:1) for 1 min at – 20°C. Later, the fixed smears were blocked with 3% bovine serum albumin (BSA) in PBS for 30 min at room temperature (RT). Mouse anti-*B. microti* P32 (rBmP32) and rabbit anti-*B. rodhaini* P26 (rBrP26) polyclonal antisera were applied as the primary antibody on the fixed smears and incubated at 37°C for 1 h in a moist chamber. After washing three times with PBS and rinsing with distilled water, Alexa Fluor^®^ 594-conjugated goat anti-rabbit IgG or Alexa Fluor^®^ 488-conjugated anti-mouse IgG (Thermo Fisher Scientific, Massachusetts, USA) was applied as the secondary antibody (diluted 1:200 in 3% BSA in PBS) on the smears, which were incubated at 37°C for 30 min. The slides were then washed three times and incubated with 200 µg/mL Hoechst 33342 solution (Thermo Fisher Scientific) diluted in 3% BSA in PBS containing 50 mg/mL RNase (Qiagen, Hilden, Germany) at 37°C for 10 min. After washing with PBS twice, the glass slides were mounted by adding 10 mL of a 50% glycerol–PBS (v/v) solution and covered with a glass coverslip. The slides were examined and micrographs were taken using the All-in-One BZ-9000 microscope (Keyence, Illinois, USA).

### Immunophenotyping of Splenocytes by Fluorescence-Activated Cell Sorting (FACS) Analysis

After surgically removing the spleen tissues from naive and infected mice in a sterile hood, splenocytes were isolated by obtaining single cell suspensions. Tissues were ground into small pieces and strained through a sterile 70 µm cell strainer in 50-mL tubes. Crude splenocyte suspensions were rinsed twice with cold 1× PBS and centrifuged at 375 × g for 5 min at 4°C. The cell pellets were lysed with 1× ACK lysis buffer (Gibco, Massachusetts, USA) for 5 min at 25°C. Cold PBS was added to the homogenates to stop the lysis, then the samples were centrifuged at 375 × g for 5 min at 4°C. The cell pellets were resuspended in 2 mL of cell staining buffer (BioLegend, California, USA) and kept on ice until staining. The viability of cells was calculated after staining with trypan blue (1:5 dilution) with the use of a hemocytometer.

Approximately one million splenocytes were reconstituted in cell staining buffer (CSB) and centrifuged. The cells were resuspended with 70 µL CSB containing CD16/CD32 monoclonal antibody for Fc blocking (Invitrogen, Massachusetts, USA) at 4°C for 25 min. Then, splenocytes were stained by labeling with respective marker antibodies conjugated with different fluorophores for 30 min at 4°C and kept in the dark. The antibody panel used in the present study is shown in **Supplementary Table 1**. Cell samples were fixed with 200 µL 4% paraformaldehyde (PFA) solution for 15 mins, washed twice with CSB, and centrifuged at 375 × g for 5 min at 4°C. Lastly, samples were resuspended in 200 µL CSB. Labeled cells were sorted using the CytoFLEX flow cytometer (Beckman Coulter, California, USA) and data was analyzed using the CytExpert 2.4 software (Beckman Coulter). A sample of the gating scheme used is depicted in **Supplementary Figure 1**, following the strategy of Bayne and Vonderheide (2013).

### Spleen Histopathological Analysis

Spleen samples were fixed in 10% neutral buffered formalin. Later, tissue samples were dehydrated through a series of graded alcohol, embedded in paraffin, and sectioned at a thickness of 4  µm. After sections were deparaffinized, staining of tissue sections was performed with hematoxylin & eosin (H & E). The slides were mounted on MGK-S (Matsunami Glass Ind. Ltd., Osaka, Japan) and covered with coverslips. A histopathologist observed changes using a Microphot-FX (Nikon) in a blinded manner. Microphotographs were taken using a Digital Sight DS-5M camera (Nikon) equipped with a microscope.

### Quantification of Serum Cytokines

Mice were exsanguinated to collect serum at days 2, 4, and 6 post-challenge infection with *B. rodhaini*. Cytokines were detected and quantified using commercial enzyme-linked immunosorbent assay (ELISA) kits (Thermo Fisher Scientific), while reactive oxygen species (ROS) levels were determined using OxiSelect™ *In Vitro* ROS/RNS Assay Kit (Cell Biolabs, Inc., California, USA), following the manufacturers’ protocols. Serum samples were diluted in PBS to 1:1,000 for cytokine detection. Absorbance values were measured using the MTP-500 microplate reader (Corona Electric Co., Tokyo, Japan), while fluorescence (480 nm excitation/530 nm emission) was read with the GloMax^®^-multi detection system (Promega, Wisconsin, USA). Obtained standard curves were extrapolated to determine the concentrations of serum IFN-γ, TNF-α, IL-2, IL-6, IL-10, IL-12, and ROS.

### Humoral Response Determination

Previously established ELISAs based on GST-fused rBmP32 and rBrP26 antigens were used to determine antibody response against each parasite species (Li et al., 2012). Microtiter plates (Nunc, Roskilde, Denmark) were coated with 0.1 µM of rBmP32 or rBrP26 and incubated at 4°C overnight. The plates were washed once with 0.05% Tween 20-PBS (PBST), then incubated with 100 µL/well of blocking solution (3% skim milk in PBS) for 1 h at 37°C. After washing, the antigen-coated plates were incubated for 1 h at 37°C with 50 µL of mouse serum diluted at 1:100 in blocking solution. Plates were washed with PBST six times and incubated for 1 h at 37°C with HRP-conjugated secondary antibody goat anti-mouse immunoglobulins IgG1, IgG2a, IgG2b, IgG2c, IgG3, IgM, and IgG diluted to 1:4,000 in blocking solution. The plates were washed again six times as described earlier, then 100 µL/well substrate solution (100 µM citric acid, 200 µM sodium phosphate, 0.3 mg of ABTS [2,2=azinobis (3-ethylbenzthiazolinesulfonic acid)]/mL (Sigma, St. Louis, MO], 0.01% of 30% H_2_O_2_) was added. After incubation for 1 h at RT, the optical density values were measured by MTP-500 microplate reader (Corona Electric) at a wavelength of 415 nm. Each serum sample was run in duplicates.

### Absolute Quantification of *B. rodhaini* Parasites

After exsanguination of mice, approximately 100 µL of blood was collected by cardiac puncture (n=4 per group), diluted in PBS (1:1), and was used to extract DNA using QIAamp^®^ DNA Blood Mini Kit (Qiagen, Hilden, Germany), following the manufacturer’s protocol. A newly designed *B. rodhaini*-specific primer set (F: 5’- CCAGGTCATTGATAACGAAGC-3’; R: 5’-TAACACCACTCATAGCGGCA-3’), amplifying a 113 bp-long *β-tubulin* gene sequence, was used to quantify *B. rodhaini* in the DNA samples collected at day 4 post-challenge infection (pci). Briefly, the reaction consisted of 5 µL 2 × PowerUp^™^ SYBR^™^ Green Master Mix (Applied Biosystems, Massachusetts, USA), 0.6 µM primers, 1 µL DNA, and distilled water up to 10 µL volume. The cycling condition used was 50°C for 2 min, 95°C for 2 min, followed by 40 cycles of 95°C for 15 sec and 60°C for 1 min, and a dissociation stage. The reactions were run in the ABI 7900HT Real-time PCR System (Applied Biosystems). Serially diluted plasmids were used to establish the standards, from which parasite count was calculated from the mean quantification cycle values of duplicated samples. Parasite numbers were transformed to log values before performing statistical analysis.

### Statistical Analyses

Comparison among groups was performed using ordinary one-way analysis of variance (ANOVA) with Tukey’s test for multiple comparisons in GraphPad Prism 8 (GraphPad Software Inc., California, USA), reported as mean ± standard deviation (SD) of 4-6 mice and are designated as follows: *p < 0.05, **p < 0.01, and ***p < 0.001. Survival curves were calculated by Kaplan-Meier non-parametric model.

## Results

### Survival of Mice With Acute *B. microti* Infection Against *B. rodhaini* Lethal Challenge

Infection with *B. rodhaini* is associated with high parasitemia and lethality in mice. The combination of different *Babesia* species resulted in different disease outcomes following infection. All mice infected with *B. rodhaini* alone (Br) and those challenged 2 days after *B. microti* primary infection (bm/br2) succumbed at day 8 pci, while complete survival was recorded from bm/br4 and bm/br6 groups (**Figure 1B**).

### Parasitemia Levels and Hematologic Indices During Acute Stage of *Babesia* Co-Infection

Usual signs of mice infected with *B. rodhaini*, including severe intravascular hemolysis linked with markedly elevated parasitemia levels (Chiou et al., 2014), were noted in this study. Throughout the course of the infection trial, parasitemia levels were monitored by examining Giemsa-stained blood smears of each mouse. At day 0 of challenge infection, the *B. microti* parasitemia for bm/br2, bm/br4, bm/br6 group was 0.087%, 0.86%, and 1.03%, respectively (**Supplementary Figure 2**). A steady rise in parasite burden was observed in all infected groups during the first week of *B. rodhaini* infection, which eventually declined after reaching the peak. The peak parasitemia levels in Br, bm/br2, and bm/br4 mice were 54.36%, 53.98%, and 47.72%, respectively, and were recorded at day 6 pci. On the other hand, the peak parasitemia level in the bm/br6 group (37.19%), which was observed two days earlier compared with those of Br, bm/br2, and bm/br4 groups, was significantly lower (**Figure 1C**). In survival groups bm/br4 and bm/br6, the lowest average body weight (20.48 ± 1.68 g) for the bm/br4 group was seen at day 8 pci, in contrast to the increased average body weight (23.79 ± 1.23 g) seen in bm/br6 group (**Figure 1D**). In addition, mice from all groups experienced anemia as characterized by their below normal hematocrit values and low RBC densities after *B. rodhaini* challenge infection (**Figures 1E, F**), with the bm/br4 group showing the most severe anemia at day 6 pci. Interestingly, peak parasitemia at day 6 pci coincided with the recorded lowest hematocrit values in bm/br2, bm/br4, and Br groups, with only the bm/br4 mice surviving two days after (day 8 pci). Normal hematocrit and RBC values were restored and maintained until day 28 pci in survival groups bm/br4 and bm/br6. Our findings confirmed that elevation in parasitemia facilitated the lysis of RBCs and decreased hematocrit values, eventually leading to hemolytic anemia, a hallmark of babesiosis.

**Figure 2:**
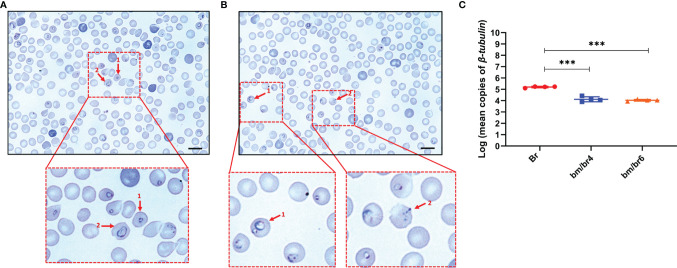
Giemsa-stained thin blood smear showing **(A)** 1, *B*. *microti*; 2, *B. rodhaini* infecting mouse erythrocytes and **(B)** 1, *B. microti* and *B. rodhaini* infecting the same erythrocyte; 2, crisis form of *Babesia*. Bars represent 10 μm. **(C)** Mean copy numbers of *B. rodhaini β-tubulin* in mouse DNA samples (n = 4 per group at day 4 post-challenge infection) were transformed to log values. Individual values are the mean of duplicate samples. Log values were analyzed using one-way ANOVA and Tukey’s multiple comparison *post-hoc* test; ***p < 0.001.

Differentiation between *Babesia* species was challenging during the different developmental stages of the parasite, except during the ring stage, where a distinct difference in the size of the ring forms of *B. microti* and *B. rodhaini* was observed (**Figures 2A, B**). During the peak parasitemia period, parasites were seen as rings within erythrocytes (**Figure 2A**), with multiple parasites also seen within a single erythrocyte (**Figure 2B**). In contrast, a large population of intraerythrocytic parasites appeared to be degenerated (**Figure 2B**). Historically, such phenotype, referred to as “crisis forms”, gets emphasized during the intraerythrocytic parasitic death (Skariah et al., 2017). At day 4 pci, significantly lower *B. rodhaini* numbers were detected in co-infected bm/br4 (30.82% parasitemia) and bm/br6 (37.19% parasitemia) groups compared with Br (9.21% parasitemia), indicating that majority of the infected RBCs were parasitized by *B. microti* (**Figure 2C**). Likewise, IFA micrographs confirmed these findings, as *B. microti* (stained in green) was the predominant species in co-infected groups (**Supplementary Figure 3A**). We also noted that the majority of the uninfected RBC population were the Hoechst-stained reticulocytes (**Supplementary Figure 3**).

### Effects of *B. microti* and *B. rodhaini* Infection on Splenic Immune Cell Population at the Acute Phase of Co-Infection

**Figure 3:**
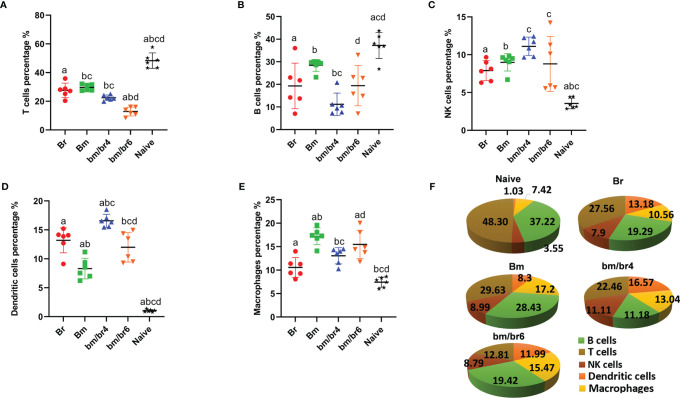
Fluorescence-activated sorting (FACS) analysis of splenic immune cells of mice at day 6 post-challenge infection. Percentage population of each cell type is presented as mean ± SD: **(A)** CD45^+^ CD3^+^ cells (T cells), **(B)** CD45^+^ CD19^+^ cells (B cells), **(C)** CD45^+^ CD49b^+^ cells (natural killer cells), **(D)** CD45^+^ CD11c^+^ cells (dendritic cells), and **(E)** CD45^+^ F4/80^+^ cells (macrophages). **(F)** Pie chart representing the population composition of T cells, B lymphocytes, NK cells, dendritic cells, and macrophages in Naive, Br, Bm, bm/br4, and bm/br6 groups. Different letters denote significant differences between groups.

In order to examine the effect of co-infection on the population of splenocytes involved in immune response and pathogenesis during the acute phase of infection, FACS analysis was performed at day 6 pci, considering it to be the peak parasitemia for Br and bm/br4 groups. Analysis of splenic immune cells showed variation in numbers of B cells, T cells, macrophages, natural killer (NK) cells, and dendritic cells (DC) (**Supplementary Table 2**). *Babesia* infection, regardless of the infection type and species, was significantly associated with significant reduction in the percent population of T cells (**Figure 3A**) and B cells (**Figure 3B**) and significant increase in the percent population of NK cells (**Figure 3C**) and DCs (**Figure 3D**). Notably, macrophages in co-infected spleen showed significantly higher populations compared to those with a single infection of *B. rodhaini* and uninfected mouse spleen (**Figure 3E**). In summary, the adaptive (T and B cells) and innate immune cell (NK, DCs, and macrophages) populations in infected mouse spleen were decreased and increased, respectively (**Figure 3F**).

### Downregulation of Pro-Inflammatory Cytokines and Upregulation of Anti-Inflammatory Cytokines During Acute Phase of *Babesia* Co-Infection

**Figure 4:**
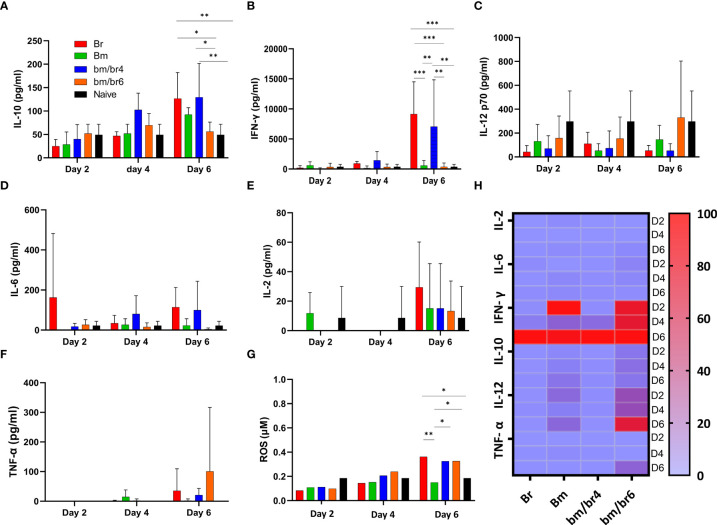
The kinetics of serum cytokines of protected and susceptible mice after *B. rodhaini* challenge infection. Test mice were initially infected with *B. microti* and challenge infected with *B. rodhaini* on days 4 and 6. On day 6 post-challenge infection, serum was collected from all groups and levels of **(A)** IL-10, **(B)** IFN- γ, **(C)** IL-12p70, **(D)** IL-6, **(E)** IL-2, and **(F)** TNF-α were measured. **(G)** ROS levels were determined by measuring serum levels of H_2_O_2_. **(H)** Heatmap showing the progression of secretion of the six cytokines at timepoints day 2 (reference), 4, 6 pci. The results are expressed as means ± SD. Ordinary one-way analysis of variance (ANOVA) with Tukey’s test was used for the statistical analysis. Asterisks indicate statistical significance (*p < 0.05; **p < 0.01; ***p < 0.001).

Cytokines are responsible for all the symptoms and pathological manifestations during infections, and IL-10 and IFN-γ are involved to moderate this process (Medina et al., 2011). In this study, the relationship between different cytokines, especially IL-10 and IFN-γ levels, was examined and the participation of pro-inflammatory and regulatory balance during the natural immune response in *Babesia*-infected mice was observed. Serum cytokines (IFN-γ, IL-10, IL-12p70, IL-6, IL-2, TNF-α, and ROS) were measured in mice acutely infected with *B. microti* and later challenged infected with *B. rodhaini* in order to pinpoint changes in immune response at day 2, 4, and 6 pci. At day 6 pci, the IL-10 serum level in Br group was comparable to that of the bm/br4 group (**Figure 4A**), while both groups had significantly higher levels of IFN-γ (**Figure 4B**) compared with other groups. A relatively higher level of IL-12 p70 in the bm/br6 group at day 6 pci compared with other groups was recorded, albeit not significantly different (**Figure 4C**). The highest concentrations of IL-6 (**Figure 4D**), IL-2 (**Figure 4E**), and ROS (**Figure 4G**) were observed in Br group. The lack of strong TNF-α levels in serum was noted as well (**Figure 4F**). Overall, the results showed upregulation of cytokines IL-10, IFN-γ, and IL-6 and downregulation of IL-12p70, Il-2, and TNF-α (**Figure 4H**) during severe *Babesia* co-infection in mice.

### Immunomodulation of Humoral Response During Acute Phase *Babesia* Co-Infection

**Figure 5:**
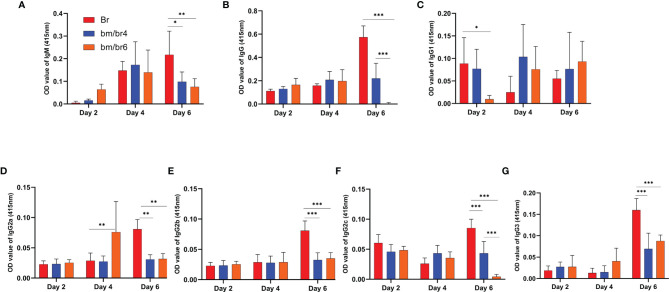
Kinetics of serum antibodies specific to *B. rodhaini* after challenge infection. The production of **(A)** IgM, **(B)** IgG, **(C)** IgG1, **(D)** IgG2a, **(E)** IgG2b, **(F)** IgG2c, and **(G)** IgG3 in mice after challenge infection with *B. rodhaini* was determined in Br mice and *B. rodhaini* and *B. microti* co-infected groups (bm/br4 and bm/br6 mice). Detection of IgGs and IgM was performed on days 2, 4, and 6. For the detection of serum antibodies against *B*. *rodhaini*, rBrP26 protein was used as the detection antigen in ELISA assays. The results are expressed as mean values ± the SD for six mice. Ordinary one-way analysis of variance (ANOVA) with Tukey’s test was used for the statistical analysis. Asterisks indicate statistical significance (*p < 0.05; **p < 0.01; ***p < 0.001).

Next, we wanted to confirm if the depletion of B and T cells (as confirmed in **Figures 3A, B**) had any effects on the species-specific antibody response; thus, antibodies against *B. rodhaini* (**Figure 5**) and *B. microti* (**Supplementary Figure 4**) were detected at day 2, 4, and 6 pci. IgM- and IgG-specific antibody responses to *B. rodhaini* challenge in mice were most pronounced at day 6 pci (**Figure 5**). IgM reacting to *B. rodhaini* antigen was detected 2 days after infection, indicating a primary immune response (**Figure 5A**), which suggests that during the early phase of acute *Babesia* infection, immune responses to parasitized erythrocytes were triggered (Chiou et al., 2014). In addition, substantial levels of IgG, IgG1, IgG3, IgG2a, IgG2b, and IgG2c were detected at day 6 pci (**Figures 5B–G**), confirming the activation of a secondary immune response against parasites.

### Changes in Splenic Immune Structure During Acute Stage of *Babesia* Co-Infection

**Figure 6:**
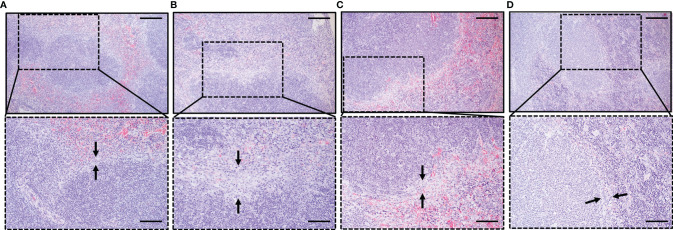
Histopathological analysis of the spleen depicting the impact of *B*. *microti*, *B*. *rodhaini*, and co-infection on the spleen compared to uninfected mice. Sections collected on day 6 post-challenge infection were stained with H & E, 100× and 200×. Black arrowheads indicate the variations in the marginal zone relative to **(A)** Naive mouse group. Erosion of the marginal zone in terms of loss of cellularity was found to be more pronounced in **(B)** Br mice compared to **(C)** bm/br4, and **(D)** bm/br6 mice. Bars in upper and lower images represent 200 and 100 μm, respectively.

In HE sections, macrophages were detected as light-stained cells by hematoxylin (blue), and the numbers increased in all groups compared with naive mouse spleen. Hence, the increase of macrophages in all groups, as revealed in **Figure 3E**, was confirmed by histopathology, and it was mostly found confined to the marginal zone. However, NK cells cannot be detected in our HE sections. We observed splenic disarray relative to the disorganized germinal center. In infected groups, disruption of the marginal zone starts during the acute phase (Djokic et al., 2018a). Erosion of the marginal zone is more pronounced during *B. rodhaini* only infection (**Figure 6B**), while less obvious during co-infection (**Figures 6C, D**), The most significant abnormality of the marginal zone was in the Br group, represented by the loss of cellularity of marginal zone (**Figure 6B**). It was characterized by increased macrophage-like cells and decreased cell density, along with the wider marginal zone as observed in the Br group than in other groups (**Figures 6A–D**).

## Discussion

To date, the immune mechanisms responsible for protection against *Babesia* are not well characterized (Aguilar-Delfin et al., 2003). Due to a co-infecting agent, an immune response to one organism may either synergistically or antagonistically induce trickle-down effects related to the infection process (Homer et al., 2000). By using a murine co-infection model (Li et al., 2012), we have described the immune response during the acute phase of *Babesia* infection. In this study, we investigated the influence of *B. microti* acute co-infection on *B. rodhaini* on the development of parasitemia, immune response, disease manifestations, hematology, the role of splenic immune effector cells, and cytokine kinetics.

At first, we aimed to confirm the previously established protection seen during *Babesia* co-infection and zoom into specific clinical and hematologic characteristics. We illustrated that *B. microti* primary infection attenuated parasitemia peak and slightly exacerbated anemia in co-infected mice. In contrast, the Br mice which received no primary infection manifested a rapid increase in parasitemia and exacerbated anemia (Wang et al., 2016). Thus, our current findings verified that the previously demonstrated cross-protection exhibited during the chronic *B. microti*-*B. rodhaini* co-infection (Li et al., 2012) also occurs and commences at a specific time point during the acute stage of co-infection. The current results demonstrate that cross-species protection against *B. rodhaini* arising from a prior *B. microti* infection can be observed in challenge-infected mice as early as day 4 post-primary infection. Within the host,* B. microti* infection exists in three phases: establishment, progression, and resolution (Knapp & Rice, 2015). After the establishment of infection, *Babesia* damages the host’s red blood cells (Dvoráková & Dvorácková, 2007). During the progression phase, damage to the erythrocytes is caused by the parasite’s egress (Sevilla et al., 2018), followed by the lysis of the host cell eventually leading to anemia and reticulocytosis (Homer et al., 2000; Kjemtrup & Conrad, 2000), which were both observed in infected mice from this study. The density of erythrocyte decreases as a consequence of the egress (Sevilla et al., 2018). It is postulated that the rupture of the plasmatic membrane of the erythrocyte is potentially accompanied by the release of hemoglobin during the course of the egress process. This may explain the hemolysis and hemoglobinuria in babesiosis (Zintl et al., 2003; Gray et al., 2010; Hildebrandt et al., 2013; Narurkar et al., 2017).

Previous studies of malaria established that parasites show tropism for mature erythrocytes. In the same way, *B. microti* also shows obvious tropism for mature erythrocytes (Borggraefe et al., 2006), as evident in the current IFA results where *B. microti* and *B. rodhaini* parasites infecting reticulocytes were rarely observed. This decline in the mature erythrocyte population during parasite infection leads to curtailed parasitemia (Okada et al., 2015), which may have contributed to the parasitemia levels plummeting right after its peak in *Babesia* co-infected mice. This tropism towards mature erythrocytes might be due to parasite-infected reticulocytes being preferably killed than those infecting mature erythrocytes (Skariah et al., 2017). Similarly, we found the remains of dead *B. microti* (crisis forms) appeared inside erythrocytes (Clark et al., 1975), as revealed by our results. Crisis forms of parasites are punctuated and degenerating parasites are induced by crisis form factor (CFF) and immune response (Sow et al., 2015). The presence of crisis forms of *Babesia* and the occurrence of reticulocytosis, with the reduced mature peripheral erythrocytes population, may be a potential mechanism for controlling *Babesia* infection.

In this study, mice infected with *B. rodhaini* produced significantly higher autoreactive IgGs compared with co-infected groups at day 6 pci, which coincided with severe hemolytic anemia. During the establishment stage, antibodies play a role in preventing erythrocyte infection either by binding the free sporozoites (Homer et al., 2000; Leitner et al., 2020) or by promoting phagocytosis of merozoites during the resolution phase, facilitating clearance of infected RBCs by phagocytic cells through a mechanism called antibody cell-dependent inhibition (ADCI) (Marsh and Kinyanjui, 2006). Severe anemia, which developed from extravascular hemolysis and enhanced erythrocyte accumulation in the spleen, may have been partially brought about by anti-erythrocyte autoantibodies. However, it is not clearly understood how these autoantibodies directly affect the pathogenesis of babesiosis (Chiou et al., 2014). Our results also showed a decline in antibody production during the acute co-infection phase, similar to a previous study (Li et al., 2012). Likewise, subversion of the adaptive immune response against *B. microti*, as seen in various co-infected *Babesia* models (Djokic et al., 2018b; Djokic et al., 2019; Efstratiou et al., 2020), may have been implicated in the specific immune response recorded in this study. In contrast, a study by Yi et al. (2018) demonstrated that despite disorganized splenic architecture during infection, *B. microti*-infected mice were able to elicit a strong adaptive immune response. It is proposed that cells of the innate immune system regulate the growth rate of the merozoites and thus, it affects the rate of parasitemia (Homer et al., 2000). Although antibodies block the invasion of merozoites, they do not play any significant part in opsonophagocytosis of *Babesia*- or *Plasmodium*-infected erythrocytes (Djokic et al., 2021).

Spleen is the prime location where regulation of detrimental immune responses take place (Bronte and Pittet, 2013). It is a vital organ involved in clearing infected RBCs, and malaria infection can result in the rupture of the spleen (Lacerda-Queiroz et al., 2017). Previous *B. microti*-*B. rodhaini* co-infected models did not provide any information regarding the analysis of spleen histopathology and splenocyte composition. Therefore, it was imperative to shed light on the situation of the spleen during the acute stage of *Babesia* co-infection. In humans and murine malaria, disorganization of splenic architecture, as well as splenomegaly, were attributed to innate immune activation, extension of monocytic cells, and elimination of infected RBCs (Weiss, 1989; Weiss et al., 1989; Villeval et al., 1990). The alterations in splenic structure, particularly the germinal centers, may affect the quality of an antibody response during malaria infection and could impact the development of immunity to malaria (Cadman et al., 2008). As *B. microti* initiates early phase activation of macrophages, this eventually leads to curbing of the replication of parasites (Terkawi et al., 2015). Subsequent studies established that *B. microti* confers immunity against other *Babesia* (Li et al., 2012) and *Plasmodium* (Efstratiou et al., 2020), which is primarily based on macrophages. Our FACS analysis results support this finding as depicted by the significant increase in splenocyte macrophage population and were confirmed by observations from the histopathology of spleen sections of co-infected mice. On the other hand, the decline in B and T cell populations noted in this study is consistent with other studies (Djokic et al., 2018a; Djokic et al., 2019). These results emphasize the pivotal role of innate immunity in the acute phase of *Babesia* co-infection in mouse models. During acute infection, there is vigorous activation of mononuclear cells resulting in apoptosis of monocytes, dendritic cells, B and T cells, leading to apoptosis of the thymus and ultimately slumping the output of naive T cells (Rénia and Goh, 2016). Contrarily, during reinfection, chronically activated T cells can induce a state of anergy and exhaustion, which may have affected the T and B cells splenocyte population in co-infected mice in the present study.

Cytokines drive the symptoms, pathological remodeling, and the consequences of the infection, which rely on the reciprocal regulation of the pro- and anti-inflammatory cytokines. Previously, *B. microti*-infected mice during the acute stage had decreased cytokine production in response to lethal *B. rodhaini* infection (Li et al., 2012). Our current findings indicate that the timing of *B. rodhaini* challenge during *B. microti* acute infection stage is crucial in the inflammatory cytokine response post-challenge, as evidenced by the cytokine levels in co-infected groups bm/br4 and bm/br6. Higher levels of cytokines could potentially allow parasite clearance during peak parasitemia (Bronte & Pittet, 2013; Djokic et al., 2018a). Thus, the role of cytokines as savior or foe in the pathogenesis of babesiosis is a time-dependent phenomenon (Ahmed, 2002).

IFN- γ (Koch et al., 2005) and IL-10 (Kumar et al., 2019), which have the ability to act as mediators of immunity, are classic examples of double-edged sword cytokines. Cross-protected mice showed lower levels of IFN- γ and IL-10, whereas Br mice showed higher levels. During blood-stage malaria, one of the pathological roles of IFN-γ is its contribution to anemia by suppressing erythropoiesis (Okada et al., 2015). As observed in both humans and mice, early production of IFN-γ leads to protection from experimental cerebral malaria (King & Lamb, 2015), while lack of early production of IFN-γ results in fatal infection (Stevenson & Riley, 2004). In both Br and bm/br4 groups, peak parasitemia and severe anemia were observed at day 6 pci. At the same time, *B. rodhaini* infection extensively damaged the spleen of mice from both groups, with dramatic reduction of cell population, especially in the marginal zone. These high pathologies may have arisen from elevated levels of circulating IFN-γ during infection. Despite comparable levels of IL-10 serum levels in Br group and bm/br4 group at day 6 pci, Br mice succumbed to the infection, while the bm/br4 group survived. IFN- γ and TNF-α overproduction is limited by IL-10 during *B. microti* infection (Djokic et al., 2018a). This scenario was also observed in our study, wherein at day 6 pci, the elevated IL-10 levels may have contributed to the relatively lower levels of IFN- γ and TNF-α in bm/br4 compared with the Br group, which became central in the survival of co-infected mice. Similarly, lethality of malaria is thought to be due to the overproduction of IL-10 in the early stage of infection (Li et al., 2012). Regardless of the origin of IL-10, it represses the function of macrophage and dendritic cells, thereby restricting Th1 and Th2 effector responses. Additionally, the production of IL-12 by monocytes is repressed by IL-10 (D’Andrea et al., 1992), which is necessary for induction of a protective immune response against malaria and shifting to a pro-inflammatory cytokine response (Crutcher et al., 1995; Xu et al., 2001). Nonetheless, the consequence of IL-10 depends on time and location and is influenced by the type of cell producing it (Kumar et al., 2019). Thus, it is possible that in the bm/br4 group, lower production of IFN-γ at day 6 pci may have slightly lowered the parasitemia, and ultimately prevented lethality. IFN-γ is recognized as a crucial cytokine that mediates splenic cell death and prompts host lethality after *Plasmodium* infection (Lacerda-Queiroz et al., 2017). Although Br and bm/br4 both groups had proportional levels of IFN-γ, decreased loss of splenic cells in the bm/br4 group may be attributed to the cross-protection immunity conferred by *B. microti*. Notably, FACS analysis showed the lowest T cell population in the bm/br6 group (12.82%), which may explain the low levels of IFN-γ at day 6 pci as Th1 cells are one of the major sources of IFN- γ.

It was suggested that a crucial part of the response that protects from the pathogenic *Babesia* WA1 is mediated by macrophages and NK cells, probably through early production of IL-12 and IFN-γ, and induction of macrophage-derived effector molecules like NO (Aguilar-Delfin et al., 2003). IL-12 plays an indispensable role in enhancing Th-1-associated immunity (Trinchieri, 1993). Early production of IFN-γ by NK cells is dependent upon IL-12 from DCs. Additionally, early induction of IFN- γ and IL-12 mediates survival and leads to protection against babesiosis (Li et al., 2012; Efstratiou et al., 2020). Moreover, Th-1-associated immunity is important in controlling blood-stage replication of *Babesia* and *Plasmodium* parasites (Li et al., 2012; Djokic et al., 2018a). Thus, IL-12 is a potent mediator of host-defense mechanisms in various experimental malaria models (Luty et al., 2000; Malaguarnera et al., 2002). Our results corroborate previous findings wherein IL-12 levels were lowest in control Br mice, while highest in bm/br6 mice, reflecting its role in the provision of immunity against fatality.

For the activation of immune cells, intracellular ROS is one of the messengers in the signaling pathway. Ty et al. (2019) showed a mechanism wherein activation of macrophages occurs by oxidative stress leading to the release of inflammatory cytokines. On the other hand, Li et al. (2012) and Johnson et al. (1996) also proposed that macrophages may kill parasites *via* reactive oxygen and nitrogen intermediates, and depending on the quantity and site of production, they may insinuate favorable or detrimental influence on malaria. Reactive oxygen and nitrogen intermediates are shown to be responsible for the resultant crisis forms of *Plasmodium* and *Babesia*. A previous study demonstrated that inoculation of NO caused lysis of erythrocytes instead of killing *P. berghei*, concluding that *Plasmodium* parasites are protected from ROS by hemoglobin released by hemolysis, but the parasite probably contains intrinsic defense mechanisms against ROS (Sobolewski et al., 2005). Macrophages produce a huge amount of ROS and RNS triggering oxidative stress (Percário et al., 2012). However, there is uncertainty regarding the role of oxidative stress during malaria. According to some authors, oxidative stress exerts protective effects, whereas others stated that it contributes to the pathophysiology of malaria. Reactive oxygen and nitrogen species (ROS and RNS), in association with oxidative stress, are responsible for systemic complications caused by malaria. When residing in host cells, *Plasmodium* parasites are prone to elevated levels of oxidative stress. In order to defend themselves, these parasites evolved ways to evade this stress in certain ways; one of them is the antioxidant defense mechanism (Percário et al., 2012). Malaria causes the induction of a “cytokine storm” with the detection of high levels of cytokines in the bloodstream (Clark, 2007; Clark et al., 2008). The mechanism by which ROS triggers inflammation during malaria is by the xanthine oxide-produced ROS giving rise to elevated levels of IL-1β, while the parasites activate caspase-1, causing the activation of the NLRP3 inflammasome (Ty et al., 2019). Hence, a new function of extracellular ROS was proposed for the induction of inflammatory cytokines and complementing with an infectious parasite for the activation of the inflammasome in macrophages. Curbing this pathway *via* hampering ROS may open new avenues for designing anti-disease remedies where pathology is contributed by oxidative stress-driven inflammation (Ty et al., 2019). As depicted by our results, the highest levels of ROS in Br mice could have possibly backfired on the host, contrary to protecting by fighting against the parasite, resulting in lysis of erythrocytes which contributes to the pathology of *B. rodhaini*.

The current study holds some weaknesses as well. In our research, we did not perform the immunohistochemistry analysis of the spleen and immunophenotyping of immune cells in blood to corroborate the splenic cell population results, which would have further strengthened our concept about immune effector cells. Furthermore, the involvement of ROS in erythrocyte lysis needs to be confirmed. As ROS may adversely affect the kidneys, it is necessary to check how this organ is affected during babesiosis. In the backdrop of this missing information, we shall have a more accurate and deeper understanding of the immune mechanism involved during co-infection, which may be helpful to design effective vaccines in the future. The way forward is to address these shortcomings in future studies.

## Conclusions

Our study demonstrated that during co-infection of mice with *Babesia* at certain time points in the acute stage, activation of the innate immune response by *B. microti* attenuated *B. rodhaini* parasitemia and led to the survival of co-infected mice. Our findings revealed that *B. microti* undermined the adaptive immune response through decreased splenic B and T cell populations, which was mirrored by the curtailed humoral immunity against both parasites. As a consequence, Th1 immune response dominated, with IL-10 and IFN- γ appearing to play major roles in the pathology of *B. rodhaini*.

## Data Availability Statement

The original contributions presented in the study are included in the article/**Supplementary Material**. Further inquiries can be directed to the corresponding author.

## Ethics Statement

All the experimental animal procedures were carried out in strict compliance with the protocols approved by the Research Ethics Review Committee of the Obihiro University of Agriculture and Veterinary Medicine (animal experiment permit: 21-133; pathogen experiment permit: 201709-7; recombinant protein protocol: 1723-5), following the guidelines of the Standards Relating to the Care and Management of Experimental Animals promulgated by Obihiro University of Agriculture and Veterinary Medicine.

## Author Contributions

IZ, EMG, and XX designed the study. IZ and EMG performed all experiments. DK conducted the histopathological analysis. JL, SJ, ML, YL, and YH assisted in the laboratory experiments. IZ and EMG analyzed the results and drafted the manuscript. JZ and XX revised the manuscript. All authors proofread, reviewed, and approved the final version of the manuscript.

## Funding

EMG is supported by a research fellowship for young scientists from the Japan Society for the Promotion of Science (JSPS), Japan (20J20134). This work was supported by the Ministry of Education, Culture, Sports, Science, and Technology of Japan (18F18089), the JSPS Core-to-Core program, and a grant from the Strategic International Collaborative Research Project (JPJ008837) promoted by the Ministry of Agriculture, Forestry, and Fisheries of Japan.

## Conflict of Interest

The authors declare that the research was conducted in the absence of any commercial or financial relationships that could be construed as a potential conflict of interest.

## Publisher’s Note

All claims expressed in this article are solely those of the authors and do not necessarily represent those of their affiliated organizations, or those of the publisher, the editors and the reviewers. Any product that may be evaluated in this article, or claim that may be made by its manufacturer, is not guaranteed or endorsed by the publisher.

## Supplementary Material

Supplementary Figure 1 Gating scheme for fluorescence-activated sorting (FACS) analysis of immune cells. A total of 50,000 events were analyzed. Each panel is a representative image of gating for **(A)** singlets, **(B)** CD45^+^ (immune cells), **(C)** CD45^+^ F4/80^+^ (macrophages), **(D)** CD45^+^ CD11c^+^ (dendritic cells), **(E)** CD45^+^ CD19^+^ (B lymphocytes), **(F)** CD45^+^ CD3^+^ (T cells), and **(G)** CD45^+^ CD49b^+^ (natural killer cells).

Supplementary Figure 2 *Babesia microti* parasitemia levels in co-infected (bm/br2, bm/br4, and bm/br6) groups prior to challenge infection with *B. rodhaini* and parasitemia of Bm group.

Supplementary Figure 3 Immunofluorescence microscopy images of blood smears from **(A)** co-infected mouse challenged at day 4 post-primary *B. microti* infection and **(B)** mouse with *B. rodhaini* infection only. Alexa Fluor^®^ 594-conjugated goat anti-rabbit IgG (green) and Alexa Fluor^®^ 488-conjugated anti-mouse IgG (red) were used as parasite markers.

Supplementary Figure 4 Kinetics of serum antibodies specific to *B. microti* after *B. rodhaini* challenge infection. The production of **(A)** IgM, **(B)** IgG, **(C)** IgG1, **(D)** IgG2a, **(E)** IgG2b, **(F)** IgG2c, and **(G)** IgG3 in mice after challenge infection with *B. rodhaini* was determined. *Babesia microti* only infection (Bm) and *B. rodhaini* and *B. microti* co-infected groups (bm/br4 and bm/br6). Detection of IgGs and IgM was performed at days 2, 4 and 6 in all groups. For detection of serum antibodies against *B. microti*, rBmP32 protein was used as detection antigen in ELISA assays. The results are expressed as mean values ± the SD of six mice. Asterisks indicate statistical significance (**p* < 0.05; ***p* < 0.01; ****p* < 0.001).

Supplementary Table 1 Antibody panel used for sorting antibody-tagged splenocytes.

Supplementary Table 2 Estimated total count and percentage of immune cell populations. ^*^Estimated numbers obtained from the mean percentage of immune cell populations.
