# Supplementary material for: The Cross-Species Immunity During Acute Babesia Co-Infection in Mice
Source: Front Cell Infect Microbiol. 2022 May 27;12:885985. doi: 10.3389/fcimb.2022.885985 (PMC9198632; doi:10.3389/fcimb.2022.885985)
Supplement: Supplementary Figure 1 — Gating scheme for fluorescence-activated sorting (FACS) analysis of immune cells. A total of 50,000 events were analyzed. Each panel is a representative image of gating for (A) singlets, (B) CD45+ (immune cells), (C) CD45+ F4/80+ (macrophages), (D) CD45+ CD11c+ (dendritic cells), (E) CD45+ CD19+ (B lymphocytes), (F) CD45+ CD3+ (T cells), and (G) CD45+ CD49b+ (natural killer cells). [file DataSheet_1.docx]

Supplementary Material

## Supplementary Table 1. Antibody panel used for sorting antibody-tagged splenocytes.

| **Immune cell population** | **Anti-mouse antibody markers** | **Fluorophore** | **Antibody dilution (vol/vol μL)** | **Manufacturer (catalog no.)** |
| --- | --- | --- | --- | --- |
| Total leukocytes | CD45 | FITC | 0.5/200 | BioLegend (147709) |
| T lymphocytes | CD3 | PE/Cyanine7 | 1/200 | BioLegend (100219) |
| B lymphocytes | CD19 | Brilliant Violet 421^™^ | 1/200 | BioLegend (115537) |
| Macrophages | F4/80 | PE | 1/200 | BioLegend (123109) |
| Natural killer cells | CD49b | PerCP-Cy5.5 | 1/200 | BioLegend (108915) |
| Dendritic cells | CD11c | Brilliant Violet 510 ^™^ | 1/200 | BioLegend (117337) |

## Supplementary Table 2. Estimated total count and percentage of immune cell populations.

| **Group** | **Immune cell marker** | **Total count^*^** | **Percentage** | **Group** | **Immune cell marker** | **Total count^*^** | **Percentage** |
| --- | --- | --- | --- | --- | --- | --- | --- |
| Br | CD45 | 15,176 |  | bm/br4 | CD45 | 38,385 |  |
|  | F4/80 | 1,604 | 10.57 |  | F4/80 | 5,009 | 13.05 |
|  | CD11c | 2,000 | 13.19 |  | CD11c | 6,360 | 16.57 |
|  | CD19 | 2,929 | 19.30 |  | CD19 | 4,291 | 11.18 |
|  | CD3 | 4,184 | 27.57 |  | CD3 | 8,625 | 22.47 |
|  | CD49b | 1,199 | 7.91 |  | CD49b | 4,265 | 11.11 |
| Bm | CD45 | 14,682 |  | bm/br6 | CD45 | 15,868 |  |
|  | F4/80 | 2,525 | 17.20 |  | F4/80 | 2,456 | 15.48 |
|  | CD11c | 1,220 | 8.31 |  | CD11c | 1,903 | 11.99 |
|  | CD19 | 4,174 | 28.43 |  | CD19 | 3,083 | 19.43 |
|  | CD3 | 4,352 | 29.64 |  | CD3 | 2,034 | 12.82 |
|  | CD49b | 1,321 | 9.00 |  | CD49b | 1,254 | 8.79 |
| Naive | CD45 | 35,605 |  |  |  |  |  |
|  | F4/80 | 2,642 | 7.42 |  |  |  |  |
|  | CD11c | 367 | 1.03 |  |  |  |  |
|  | CD19 | 13,252 | 37.22 |  |  |  |  |
|  | CD3 | 17,201 | 48.31 |  |  |  |  |
|  | CD49b | 1,264 | 3.55 |  |  |  |  |

^*^Estimated numbers obtained from the mean percentage of immune cell populations.


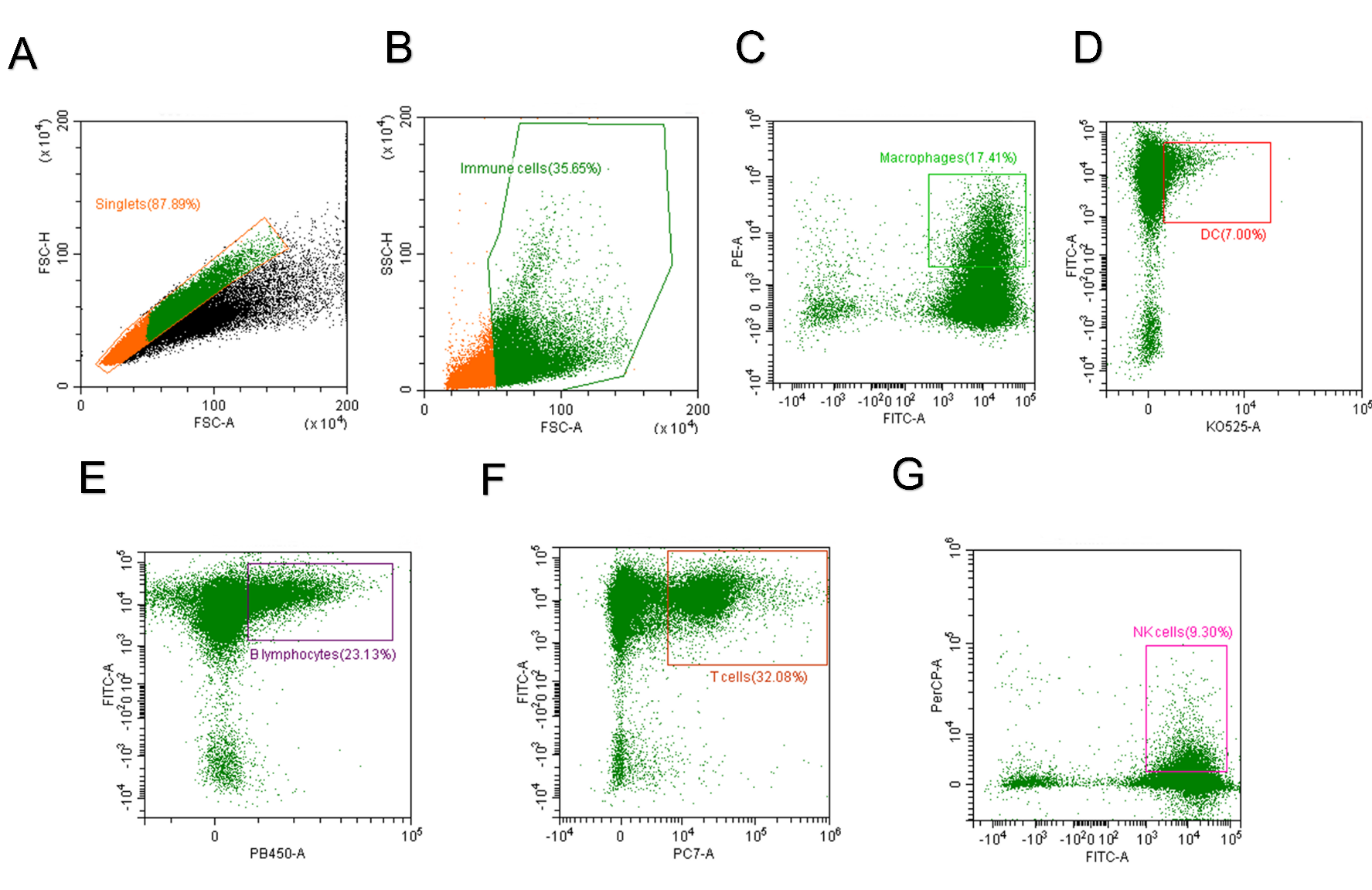


**Supplementary Figure 1.** Gating scheme for fluorescence-activated sorting (FACS) analysis of immune cells. A total of 50,000 events were analyzed. Each panel is a representative image of gating for (A) singlets, (B) CD45^+^ (immune cells), (C) CD45^+^ F4/80^+^ (macrophages), (D) CD45^+^ CD11c^+^ (dendritic cells), (E) CD45^+^ CD19^+^ (B lymphocytes), (F) CD45^+^ CD3^+^ (T cells), and (G) CD45^+^ CD49b^+^ (natural killer cells).


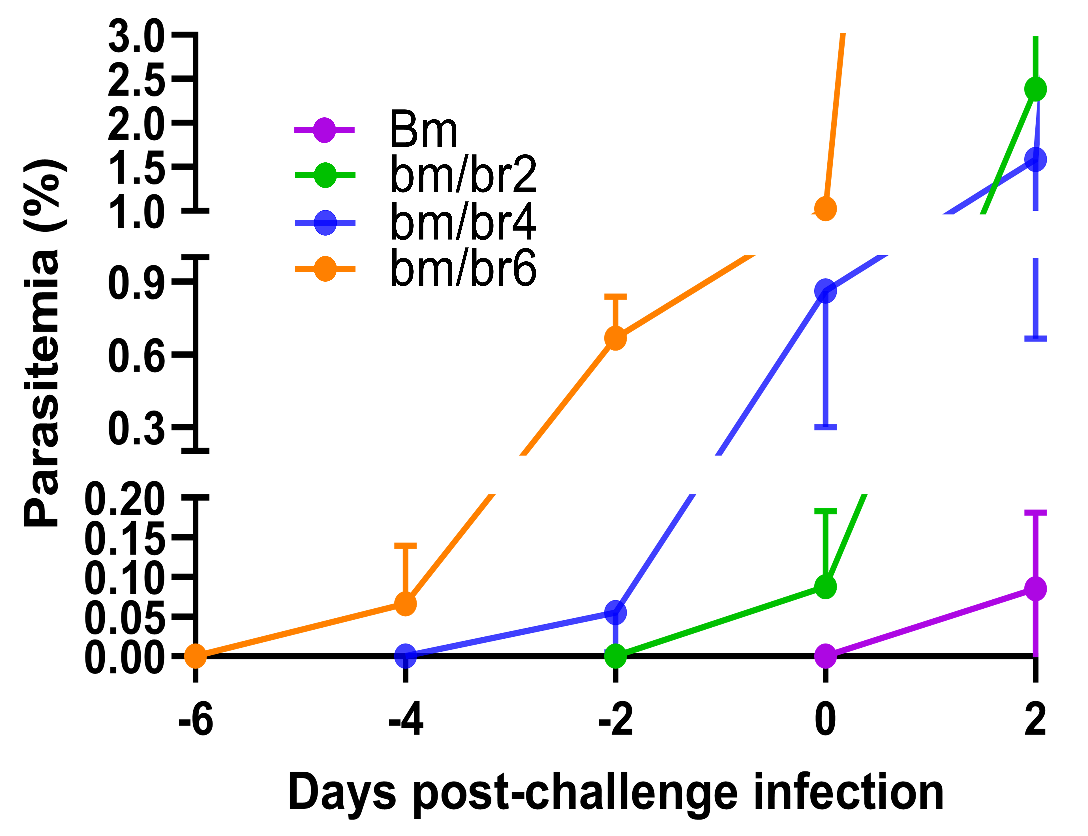


**Supplementary Figure 2.** *Babesia microti* parasitemia levels in co-infected (bm/br2, bm/br4, and bm/br6) groups prior to challenge infection with *B. rodhaini* and parasitemia of Bm group*.*


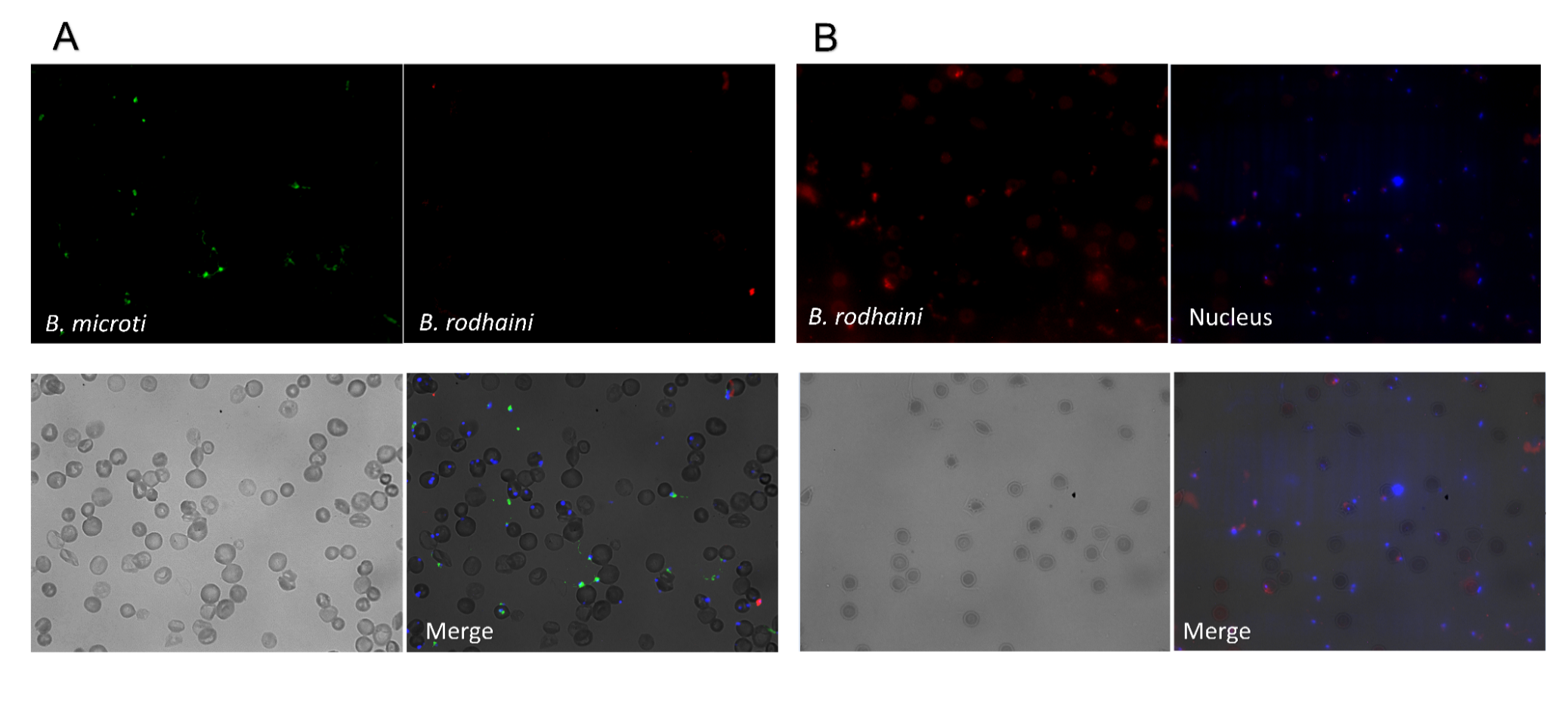


**Supplementary Figure 3.** Immunofluorescence microscopy images of blood smears from **(A)** co-infected mouse challenged at day 4 post-primary *B. microti* infection and **(B)** mouse with *B. rodhaini* infection only. Alexa Fluor^®^ 594-conjugated goat anti-rabbit IgG (green) and Alexa Fluor^®^ 488-conjugated anti-mouse IgG (red) were used as parasite markers.


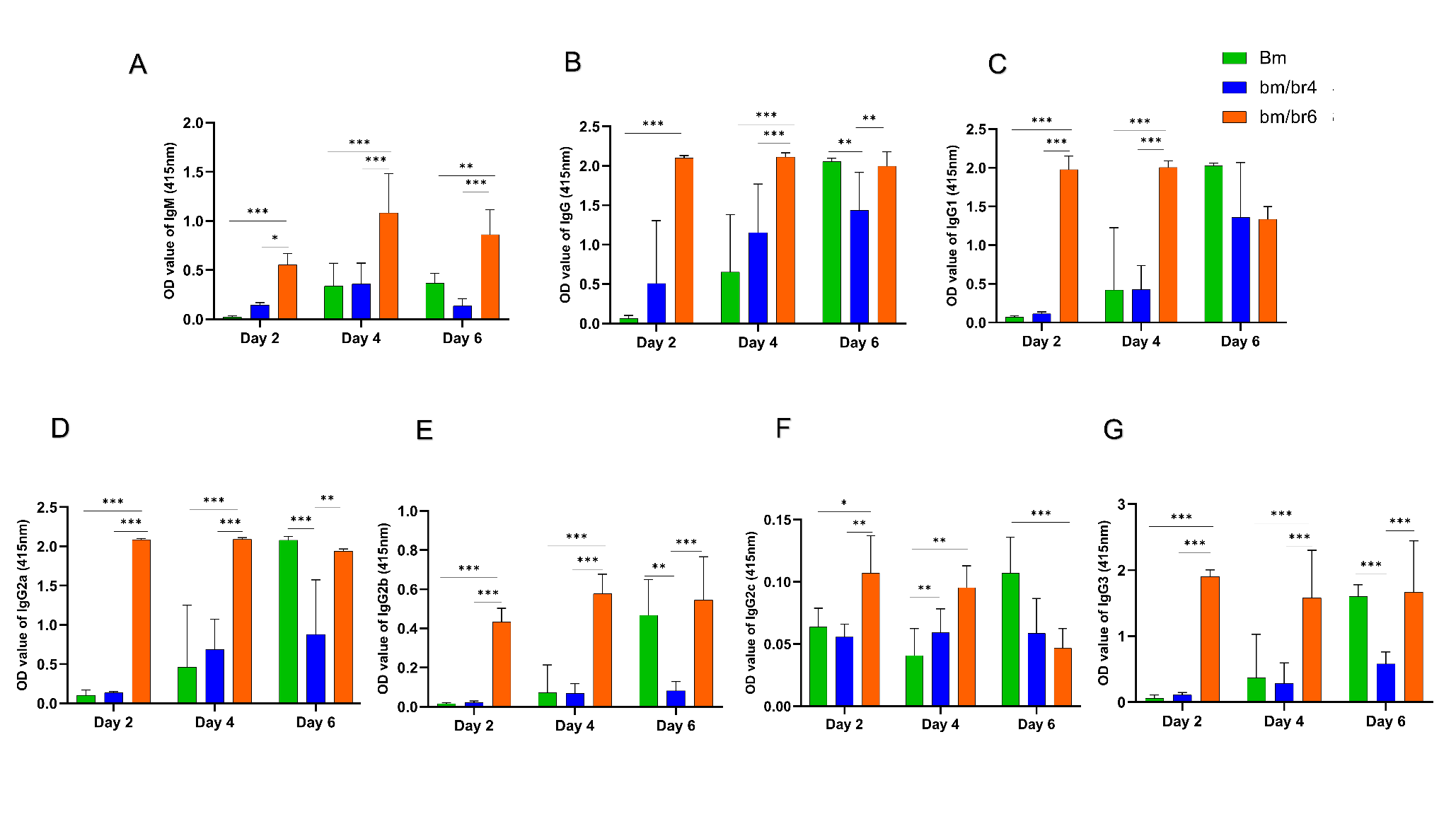
**Supplementary Figure 4.** Kinetics of serum antibodies specific to *B. microti* after *B. rodhaini* challenge infection. The production of **(A)** IgM, **(B)** IgG, **(C)** IgG1, **(D)** IgG2a, **(E)** IgG2b, **(F)** IgG2c, and **(G)** IgG3 in mice after challenge infection with *B. rodhaini* was determined. *Babesia microti* only infection (Bm) and *B. rodhaini* and *B. microti* co-infected groups (bm/br4 and bm/br6). Detection of IgGs and IgM was performed at days 2, 4 and 6 in all groups. For detection of serum antibodies against *B. microti*, rBmP32 protein was used as detection antigen in ELISA assays. The results are expressed as mean values ± the SD of six mice. Asterisks indicate statistical significance (**p* < 0.05; ***p* < 0.01; ****p* < 0.001).
